# SARS-CoV-2 Surveillance in the Middle East and North Africa: Longitudinal Trend Analysis

**DOI:** 10.2196/25830

**Published:** 2021-01-15

**Authors:** Lori Post, Emily Marogi, Charles B Moss, Robert Leo Murphy, Michael G Ison, Chad J Achenbach, Danielle Resnick, Lauren Singh, Janine White, Michael J Boctor, Sarah B Welch, James Francis Oehmke

**Affiliations:** 1 Buehler Center for Health Policy and Economics Feinberg School of Medicine Northwestern University Chicago, IL United States; 2 Feinberg School of Medicine Northwestern University Chicago, IL United States; 3 Institute of Food and Agricultural Sciences University of Florida Gainsville, FL United States; 4 Insitute of Global Health Feinberg School of Medicine Northwestern University Chicago, IL United States; 5 Division of Infectious Disease Feinberg School of Medicine Northwestern University Chicago, IL United States; 6 International Food Policy Research Institute Washington DC, DC United States

**Keywords:** COVID-19, SARS-CoV-2 surveillance, wave two, second wave, global COVID-19 surveillance, MENA public health surveillance, MENA COVID-19, Middle East and North Africa surveillance metrics, dynamic panel data, MENA econometrics, MENA SARS-CoV-2, Middle East and North Africa COVID-19 surveillance system, MENA COVID-19 transmission speed, MENA COVID-19 transmission acceleration, COVID-19 transmission deceleration, COVID-19 transmission jerk, COVID-19 7-day lag, SARS-CoV-2, Arellano-Bond estimator, generalized method of moments, GMM, Bahrain, Iran, Iraq, Israel, Jordan, Kuwait, Lebanon, Oman, Qatar, Saudi Arabia, Syria, United Arab Emirates, Yemen, Algeria, Djibouti, Egypt, Libya, Morocco, Tunisia

## Abstract

**Background:**

The COVID-19 pandemic has disrupted the lives of millions and forced countries to devise public health policies to reduce the pace of transmission. In the Middle East and North Africa (MENA), falling oil prices, disparities in wealth and public health infrastructure, and large refugee populations have significantly increased the disease burden of COVID-19. In light of these exacerbating factors, public health surveillance is particularly necessary to help leaders understand and implement effective disease control policies to reduce SARS-CoV-2 persistence and transmission.

**Objective:**

The goal of this study is to provide advanced surveillance metrics, in combination with traditional surveillance, for COVID-19 transmission that account for weekly shifts in the pandemic speed, acceleration, jerk, and persistence to better understand a country’s risk for explosive growth and to better inform those who are managing the pandemic. Existing surveillance coupled with our dynamic metrics of transmission will inform health policy to control the COVID-19 pandemic until an effective vaccine is developed.

**Methods:**

Using a longitudinal trend analysis study design, we extracted 30 days of COVID-19 data from public health registries. We used an empirical difference equation to measure the daily number of cases in MENA as a function of the prior number of cases, the level of testing, and weekly shift variables based on a dynamic panel data model that was estimated using the generalized method of moments approach by implementing the Arellano-Bond estimator in R.

**Results:**

The regression Wald statistic was significant (χ^2^_5_=859.5, *P*<.001). The Sargan test was not significant, failing to reject the validity of overidentifying restrictions (χ^2^_294_=16, *P*=.99). Countries with the highest cumulative caseload of the novel coronavirus include Iran, Iraq, Saudi Arabia, and Israel with 530,380, 426,634, 342,202, and 303,109 cases, respectively. Many of the smaller countries in MENA have higher infection rates than those countries with the highest caseloads. Oman has 33.3 new infections per 100,000 population while Bahrain has 12.1, Libya has 14, and Lebanon has 14.6 per 100,000 people. In order of largest to smallest number of cumulative deaths since January 2020, Iran, Iraq, Egypt, and Saudi Arabia have 30,375, 10,254, 6120, and 5185, respectively. Israel, Bahrain, Lebanon, and Oman had the highest rates of COVID-19 persistence, which is the number of new infections statistically related to new infections in the prior week. Bahrain had positive speed, acceleration, and jerk, signaling the potential for explosive growth.

**Conclusions:**

Static and dynamic public health surveillance metrics provide a more complete picture of pandemic progression across countries in MENA. Static measures capture data at a given point in time such as infection rates and death rates. By including speed, acceleration, jerk, and 7-day persistence, public health officials may design policies with an eye to the future. Iran, Iraq, Saudi Arabia, and Israel all demonstrated the highest rate of infections, acceleration, jerk, and 7-day persistence, prompting public health leaders to increase prevention efforts.

## Introduction

### Background

**Figure 1:**
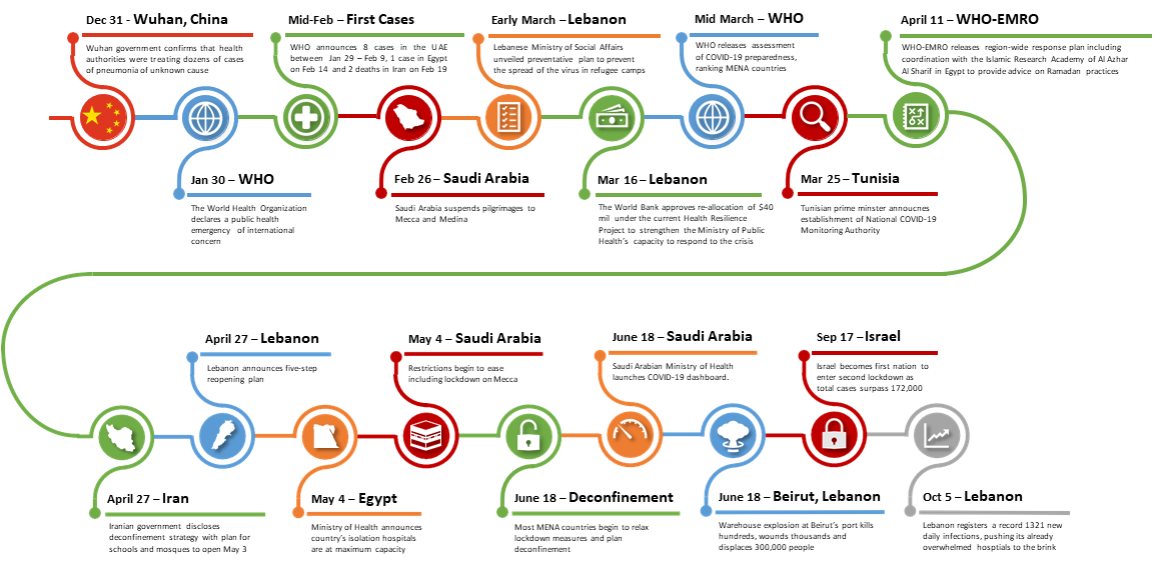
Middle East and North Africa (MENA) timeline. WHO: World Health Organization; UAE: United Arab Emirates; EMRO: Regional Office for the Eastern Mediterranean.

SARS-CoV-2, the novel coronavirus that causes the disease COVID-19, first presented in December 2019 in Wuhan City, China, and was declared a public health emergency of international concern on January 30, 2020, as it spread quickly around the globe through human-to-human transmission [1-4] . According to the World Health Organization (WHO), the first reports of COVID-19 in the Middle East and North Africa (MENA) included 8 cases in the United Arab Emirates between January 29, 2020, and February 9, 2020 [5] (Figure 1). It has since spread to every country in the region [6]. MENA is divided by large wealth disparities and regional conflicts, increasing risks to the COVID-19 pandemic that has no political or religious agenda [6]. Despite varying levels of health system preparedness in MENA countries, early implementation of strict containment measures helped to limit the spread of SARS-CoV-2. As of October 28, the WHO reported 43,540,739 confirmed cases of COVID-19, including 1,160,650 deaths worldwide [7]. There have been 2,982,597 confirmed cases in MENA, resulting in 75,737 deaths [7].

Many countries use a combination of containment and mitigation strategies, including isolation of cases, contact tracing, social distancing, border closures, masking, hand and surface hygiene, and travel restrictions [8]. In the absence of a vaccine, worldwide systemic public health surveillance that can reliably track cases and identify where outbreaks will occur is needed to inform disease control policy for COVID-19 prevention [4,9]. Global SARS-CoV-2 surveillance necessitates dividing the globe into separately surveyed regions. The World Bank, an international financial institution that aims to reduce poverty and increase sustainable prosperity in nations throughout the globe, divides the world into regions based on shared geographic, cultural, and historical qualities. The MENA region comprises Bahrain, Iran, Iraq, Israel, Jordan, Kuwait, Lebanon, Oman, Qatar, Saudi Arabia, Syria, the United Arab Emirates, Yemen, Algeria, Djibouti, Egypt, Libya, Morocco, and Tunisia [10].

Responses to COVID-19 in MENA have ranged from restrictive temporary lockdowns to denial and lack of organization [6,11]. Lessons learned from the severe acute respiratory syndrome (SARS), the Middle East respiratory syndrome (MERS), and the 2009 H1N1 outbreaks inform the current pandemic [12]. For example, in Saudi Arabia, the Ministry of Health established the Command and Control System and the Saudi Center for Disease Control and Prevention shortly after the rise of MERS [12]. Some countries repurposed surveillance resources to encourage COVID-19 containment. This is best illustrated in the United Arab Emirates where the “Oyoon” (“eyes” in Arabic) surveillance camera program, originally developed to track and prevent crime, was repurposed to check the temperatures of those passing by and to ensure people are social distancing [13].

### Economics

Countries in MENA face a dual crisis from the COVID-19 pandemic and the collapse of oil prices [14]. These concurrent crises are exacerbated by structural economic challenges including large and inefficient public sectors, uncompetitive business environments, high youth and female unemployment, governance challenges, and regional conflicts [14]. Given these challenges, the World Bank released a report recommending a coordinated regional MENA trade integration framework that lays the foundation for global value chain integration and thus helps MENA’s economic prospects [14]. Income disparities abound in MENA, which is home to the wealthy Persian Gulf nations and yet is one of the only regions in the world where extreme poverty has been rising since 2011, particularly in regions with violent conflicts [6,15]. Thus, as countries in MENA combat the COVID-19 pandemic, they do so in the context of falling oil prices and disparities in wealth and political stability.

Violent conflicts significantly weakened the health infrastructure in several countries across MENA, resulting in poor health worker capacity [12,16]. However, outside of conflict regions, MENA countries have some of the lowest proportion of health workers in the world with Egypt, Morocco, Iraq, Yemen, and Djibouti subject to low levels of doctors and nurses [11]. The health system capacity in MENA is facility based, which limits the surge capacity needed to respond to a crisis such as the COVID-19 pandemic [11]. Some MENA countries have limited public health infrastructure, including Egypt, Iraq, Djibouti, and Yemen that spend less than 5% of their government funding on health [11,17].

Middle Eastern populations have a high disease burden, which suggests a reduction in routine health service utilization results in increased mortality, a trend expected to hold true for COVID-19 [11,12]. Food insecurity poses a significant public health concern in countries with high refugee per capita concentration, such as Lebanon and Jordan [18-20]. Refugee populations are particularly vulnerable to COVID-19 as they are often concentrated in densely populated camps with poor hygiene measures and fragmented access to health care [12].

On August 4, 2020, a deadly warehouse explosion, caused by 2750 tons of ammonium nitrate stored at Beirut’s port, killed hundreds, wounded and displaced thousands, left 5 hospitals in the area either nonfunctional or only partially functional, and destroyed 17 containers of WHO essential medical supplies [21-23]. The seeming carelessness with which this destructive material was stored for 6 years fueled public outrage and antigovernment protests throughout the city [24]. Since then, Lebanon has seen a 220% increase in COVID-19 cases [25]. As countries ease restrictions and a second wave of COVID-19 threatens to undo progress, it is particularly important to accurately track cases across MENA [26]. This is evident in countries such as Israel [27]. Ideally, advanced tracking and estimation of COVID-19 transmission will inform the implementation of public health policies. Though general public health surveillance is helpful and provides a proxy of the pandemic, surveillance data suffer from significant bias due to undercounts, reporting delays, testing errors, dearth of testing, asymptomatic carriers, and other types of data contamination [7,28-35]. In fact, surveillance systems are predicated on the fact that they tend to include the more severe cases and suffer from incomplete case ascertainment by missing the mild cases and undiagnosed infections and deaths.

### Objective

The objective of our research is to provide additional surveillance metrics to add to the public health arsenal using dynamic panel modeling and method of moments to minimize sampling bias by measuring significant negative or positive weekly shifts in the increase, decrease, or plateaued transmission of SARS-CoV-2. We will apply novel indicators derived specifically to inform policy makers about the COVID-19 pandemic [3,36], including weekly shifts in the pandemic, speed, acceleration and deceleration, jerk, and 7-day persistence. These dynamic metrics will better inform public health policy when combined with standard surveillance measures.

## Methods

This study relies on a longitudinal trend analysis of data collected from the Foundation for Innovative New Diagnostics (FIND) [37]. FIND compiles data from multiple sources across individual websites, statistical reports, and press releases; data for the most recent 8 weeks were accessed from the GitHub repository [38]. This resulted in a panel of 19 MENA countries with 30 days in each panel (n=570). An empirical difference equation was specified in which the number of new positive cases in each country on each day is a function of prior numbers of cases, level of testing, and weekly shift variables that measure whether the contagion was growing faster, at the same pace, or slower than the previous weeks. This resulted in a dynamic panel data model that was estimated using the generalized method of moments (GMM) approach by implementing the Arellano-Bond estimator in R (The R Foundation) [3,36,39].

## Results

### MENA Regression Results

Regression results are presented for 17 MENA countries in Table 1. Weekly surveillance data in Tables 2-7 are based on these regressions.

The regression Wald statistic was significant (*χ*^2^_5_=859.5, *P*<.001). The Sargan test was not significant, failing to reject the validity of overidentifying restrictions (*χ*^2^_294_=16, *P*=.99)

**Table 1:** Arellano-Bond dynamic panel data modeling of the number of daily infections reported by country, October 5-18, 2020.

Variable	Statistic	*P* value
L7Pos^a^	*r*=0.57	<.001
Cumulative tests	*r*=0.00012	<.001
Weekend	*r*=0.04	.95
Wald statistic for regression	*χ*^2^_5_= 859.5	<.001
Sargan statistic for validity	*χ*^2^_294_=16	.99

^a^L7Pos: the statistical impact of the 7-day lag of speed on today’s value of speed. New cases per day tend to have an echo effect 7 days later, similar to the echo effect in the population pyramid caused by the baby boom. Reported as the weekly average number of new cases per day that are attributable to the weekly average of the 7-day lag of the number of new cases per day.

Tables 2 and 3 contain standard surveillance metrics for the weeks of October 5-11 and October 12-18. These metrics include new daily COVID-19 cases, cumulative COVID-19 cases, 7-day moving averages, rate of infection, new deaths, cumulative deaths, 7-day moving average of death rates, and rates of death per 1,000,000 population.

**Table 2:** Static surveillance metrics for the week of October 5-11, 2020.

Country	New COVID-19 cases, n	Cumulative COVID-19 cases, n	7-day moving average of new cases	Rate of infection	New deaths, n	Cumulative deaths, n	7-day moving average of death rate	Rate of death
Algeria	132	53,072	133.71	0.31	6	1801	5.86	0.01
Bahrain	327	75,614	421.71	19.92	2	275	2.14	0.12
Djibouti	0	5423	0.57	0.00	0	61	0.00	0.00
Egypt	129	104,516	119.00	0.13	12	6052	10.14	0.01
Iran	3822	500,075	4043.29	4.61	251	28,544	226.71	0.30
Iraq	2206	402,330	3312.71	5.61	62	9852	64.71	0.16
Israel	618	290,493	3388.29	6.83	39	1980	37.29	0.43
Jordan	928	24,926	1326.57	9.19	10	191	12.86	0.10
Lebanon	1010	53,568	1298.00	14.73	4	459	7.57	0.06
Libya	1026	42,712	843.29	15.14	8	631	5.57	0.12
Morocco	2563	152,404	2733.14	7.03	33	2605	39.29	0.09
Oman	1761	105,890	660.00	35.40	29	1038	8.71	0.58
Qatar	207	127,985	212.43	7.31	1	220	0.57	0.04
Saudi Arabia	323	339,267	411.43	0.94	25	5043	24.00	0.07
Tunisia	1297	32,556	1475.14	11.09	22	478	22.43	0.19
United Arab Emirates	1096	106,229	1061.14	11.22	2	445	2.71	0.02
Region	17,445	2,417,060	18,569.14	4.35	506	59,675	470.57	0.13

**Table 3:** Static surveillance metrics for the week of October 12-18, 2020.

Country	New weekly COVID-19 cases, n	Cumulative COVID-19 cases, n	7-day moving average of new cases	Rate of infection	New weekly deaths, n	Cumulative deaths, n	7-day moving average of death rate	Rate of deaths per 100k
Algeria	199	54,402	190	0.46	10	1856	7.86	0.02
Bahrain	331	77,902	326.86	20.17	7	300	3.57	0.43
Djibouti	7	5459	5.14	0.72	0	61	0.00	0.00
Egypt	127	105,424	129.71	0.13	11	6120	9.71	0.01
Iran	3890	530,380	4329.29	4.69	252	30,375	261.57	0.30
Iraq	3110	426,634	3472	7.91	56	10,254	57.43	0.14
Israel	339	303,109	1802.29	3.74	19	2209	32.71	0.21
Jordan	1520	37,573	1806.71	15.05	15	345	22.00	0.15
Lebanon	1002	62,286	1245.43	14.62	3	520	8.71	0.04
Libya	945	48,790	868.29	13.94	26	725	13.43	0.38
Morocco	2721	173,632	3032.57	7.46	50	2928	46.14	0.14
Oman	1657	109,953	580.43	33.31	30	1101	9.00	0.60
Qatar	204	129,431	206.57	7.20	1	224	0.57	0.04
Saudi Arabia	348	342,202	419.29	1.02	20	5185	20.29	0.06
Tunisia	0	40,542	1140.86	0.00	0	626	21.14	0.00
United Arab Emirates	1215	115,602	1339.00	12.44	4	463	2.57	0.04
Region	17,615	2,563,321	18,397.14	4.39	504	63,292	516.71	0.13

In Table 3, we see the countries with the highest cumulative caseload of the novel coronavirus, which includes Iran, Iraq, Saudi Arabia, and Israel with 530,380, 426,634, 342,202, and 303,109 cases, respectively. To eliminate the week-end effect or delayed reporting, we look at 7-day moving average to understand the 7-day average number of cases. In the second week of our study, October 12-18, Iran reported 4329, Israel reported 1802, Iraq reported 3472, and Morocco reported 3033 new cases per day (weekly averages). The next closest country in terms of average new cases only has a third of those cases. We find that many of the smaller countries in MENA have higher infection rates than those countries with the highest caseload. For example, Oman had 33.3 new infections per 100,000 population while Bahrain had 12.1, Libya had 14, and Lebanon had 14.6. In order of most to least number of cumulative deaths since January 2020, Iran, Iraq, Egypt, and Saudi Arabia had 30,375, 10,254, 6120, and 5185, respectively. These countries are outliers when comparing them to the rest of the region, which all have less than 3000 cumulative deaths

**Figure 2:**
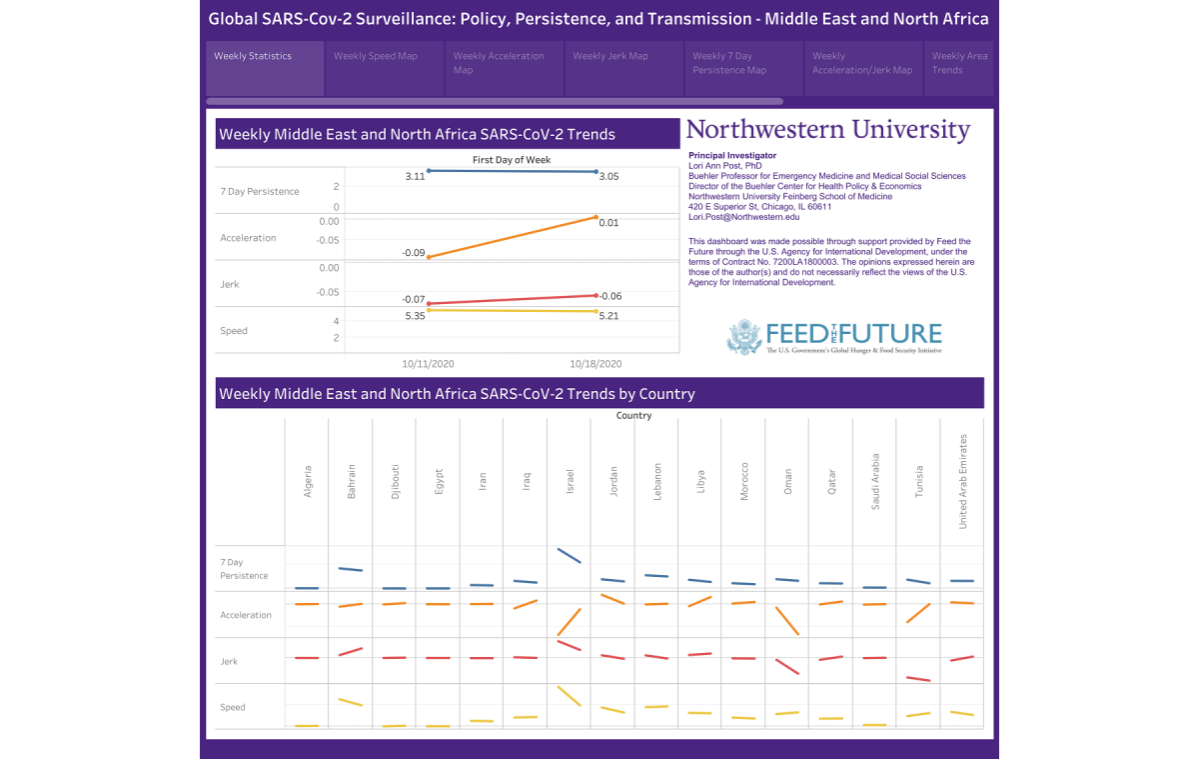
Middle East and North Africa weekly trends [40].

The novel surveillance metrics are presented in Tables 4 and 5 and Figure 2 [40] and Multimedia Appendices 1-5.

**Table 4:** Novel surveillance metrics for the week of October 5-11, 2020.

Country	Speed^a^	Acceleration^b^	Jerk^c^	7-day persistence effect on speed^d^
Algeria	0.31	0.00	0.00	0.20
Bahrain	25.70	–0.22	0.48	16.37
Djibouti	0.06	–0.01	0.00	0.08
Egypt	0.12	0.00	0.00	0.07
Iran	4.88	0.03	–0.03	2.49
Iraq	8.43	–0.36	0.12	6.15
Israel	37.43	–2.70	2.83	32.14
Jordan	13.13	0.05	–0.14	5.76
Lebanon	18.93	0.05	–0.09	9.77
Libya	12.44	0.64	0.75	5.34
Morocco	7.49	0.20	–0.10	3.48
Oman	13.27	–2.65	–2.65	6.25
Qatar	7.50	0.24	0.23	4.06
Saudi Arabia	1.20	–0.03	–0.02	0.76
Tunisia	12.61	0.01	–3.82	4.26
United Arab Emirates	10.86	0.08	0.23	6.11
Region	5.35	–0.09	–0.07	3.11

^a^Daily positives per 100k (weekly average of new daily cases per 100k).

^b^Day-to-day change in the number of positives per day, weekly average, per 100k.

^c^Week-over-week change in acceleration, per 100k.

^d^New cases per day per 100k attributed to new cases 7 days ago.

**Table 5:** Novel surveillance metrics for the week of October 12-18, 2020.

Country	Speed^a^	Acceleration^b^	Jerk^c^	7-day persistence effect on speed^d^
Algeria	0.44	0.02	0.00	0.18
Bahrain	19.92	0.03	1.61	14.64
Djibouti	0.53	0.10	0.06	0.03
Egypt	0.13	0.00	0.00	0.07
Iran	5.22	0.01	–0.03	2.78
Iraq	8.83	0.33	0.01	4.80
Israel	19.91	–0.44	1.36	21.32
Jordan	17.89	0.84	0.46	7.48
Lebanon	18.17	–0.02	0.44	10.79
Libya	12.81	–0.17	0.50	7.09
Morocco	8.31	0.06	–0.06	4.27
Oman	11.67	–0.30	–0.30	7.56
Qatar	7.29	–0.02	–0.30	4.27
Region	5.21	0.01	–0.06	3.05
Saudi Arabia	1.22	0.01	0.03	0.68
Tunisia	9.76	–1.58	–3.28	7.19
United Arab Emirates	13.70	0.17	–0.42	6.19
Region	5.21	0.01	–0.06	3.05

^a^Daily positives per 100k (weekly average of new daily cases per 100k).

^b^Day-to-day change in the number of positives per day, weekly average, per 100k.

^c^Week-over-week change in acceleration, per 100k.

^d^New cases per day per 100k attributed to new cases 7 days ago.

Overall, the MENA region was more stable than other global regions, but metrics between October 5-18 indicate pending growth. The speed of new infections decelerated during the week of October 5-11 and remained stable during the week of October 11-18. In addition, in the latter week, there was a slight negative jerk. The persistence rate slightly decreased from 3.11 to 3.05 per 100,000 population over the study period, which directly measures those new cases that are statistically related to the number of new infections 7 days earlier. While the surveillance metrics for the MENA region as a whole are promising, they are averages and thus we must look to those countries with increasing rates of speed, acceleration, jerk, and persistence to understand which countries have outbreaks and less control of the pandemic. In Table 5, we note that Bahrain, Israel, and Lebanon decreased in speed or remained level between October 5 and 18. Jordan and the United Arab Emirates had significant increases in rates of infection. Tunisia had the best shift in the pandemic that resulted in a decrease in speed week over week. In addition, the rates reversed course and decelerated from October 5-11 to October 12-18, and Tunisia experienced a negative jerk in infection rates 2 weeks in a row, indicating a strong downward trend in COVID-19 infections. For Israel, the 7-day persistence effect for the week of October 12-18 (21 new cases) slightly overpredicts the speed or the actual number of new cases (n=20); coupled with a declining persistence rate relative to the prior week of October 5-11, this is indicative of a rapid descent from a recent period of explosive growth. On the other end of the spectrum, Bahrain had positive speed, acceleration, and jerk, indicating the outbreak is increasing and is leading toward a worsening pandemic.

**Table 6:** Seven-day persistence difference.

Country	7-day persistence	
	October 5-11, 2020	October 12-18, 2020
Israel	32.14	21.32
Bahrain	16.37	14.64
Lebanon	9.77	10.79
Oman	6.25	7.56
Iraq	6.15	—^a^
Jordan	—	7.48

^a^Not applicable.

Israel, Bahrain, Lebanon, and Oman had the highest rate of COVID-19 persistence, which is the number of new infections statistically related to new infections from 7 days ago. These 3 countries had higher persistence rates 2 weeks in a row. Finally, Egypt has the highest population in MENA (Table 7) and the potential for growth but has remained stable across the 2 weeks of this study, indicating that COVID-19 is not accelerating.

**Table 7:** Most populous countries in the Middle East and North Africa.

Country	Population as of 2020, n
Egypt	102,334,404
Iran	83,992,949
Algeria	43,851,044
Iraq	40,222,493
Morocco	36,910,560

## Discussion

### Principal Findings

Analysis at the country level indicates there are some nations that should increase their public health efforts to gain control of the COVID-19 pandemic. Iran, Israel, Iraq, and Morocco had the highest reported 7-day average per 100,000 population, which is significantly higher than other nations in the region. Iran, Iraq, Saudi Arabia, and Israel had the highest caseload at the end of October 18. Looking toward the future, Jordan, Iraq, and the United Arab Emirates have the fastest acceleration in new COVID-19 infections while Bahrain and Israel have the largest upwards jerk in infections, which can lead to explosive growth. Iran began the pandemic with explosive growth but during the last week of this study ending on October 18, Iran’s acceleration rate has leveled off and its jerk has reversed lower; however, given the number of new cases and population size, the country could easily flare up in new outbreaks, especially considering the second wave of COVID-19 infections has just begun.

### Limitation

Data are limited by granularity and collection method. Data were collected at the country level, which precludes local analysis of surveillance trends. Moreover, data collection mechanisms differ by country and may even differ by region within a given country. These different methods lead to week-end effects, missing data points, and other contamination.

### Comparison With Prior Work

This study is part of a broader research program at Northwestern University Feinberg School of Medicine, *The Global SARS-CoV-2 Surveillance Project: Policy, Persistence, & Transmission*. This research program developed novel surveillance metrics to include rates of speed, acceleration, jerk, and 7-day persistence [3,36]. We have also derived surveillance metrics for all global regions.

### Conclusion

Static and dynamic public health surveillance tools provide a more complete picture of pandemic progression across countries and regions. While static measures capture data at a given point in time, like infection rates and death rates, they are less successful at assessing population health over a period of weeks or months. By including speed, acceleration, jerk, and 7-day persistence, public health officials may design policies with an eye to the future.

MENA countries with the highest risk all shared a number of characteristics according to the surveillance data. There was a definite positive shift between October 5-11 and October 12-18. Iran, Iraq, Saudi Arabia, and Israel all demonstrated the highest numbers of cumulative infections, acceleration, jerk, and 7-day persistence rates. Looking ahead, policy makers in these countries and the region at large should be concerned about growth in the already substantial number of cases over the short term. Given the substantial 7-day persistence rates of Israel, Bahrain, and Lebanon, it is imperative that efforts be made to target super spreader events. Analysis of subsequent surveillance data using both static and dynamic tools can help confirm the efficaciousness of new policies.

## Appendix

Multimedia Appendix 1 Weekly MENA country statistics.

Multimedia Appendix 2 Weekly MENA SARS-CoV-2 trends.

Multimedia Appendix 3 Weekly MENA acceleration and jerk map.

Multimedia Appendix 4 Weekly MENA 7-day persistence map.

Multimedia Appendix 5 Weekly MENA speed map.

## Abbreviations

FIND: Foundation for Innovative New Diagnostics
GMM: generalized method of moments
MENA: Middle East and North Africa
MERS: Middle East respiratory syndrome
SARS: severe acute respiratory syndrome
WHO: World Health Organization
