# Supplementary figures and images for: SARS-CoV-2 Surveillance in the Middle East and North Africa: Longitudinal Trend Analysis
Source: J Med Internet Res. 2021 Jan 15;23(1):e25830. doi: 10.2196/25830 (PMC7813562; doi:10.2196/25830)

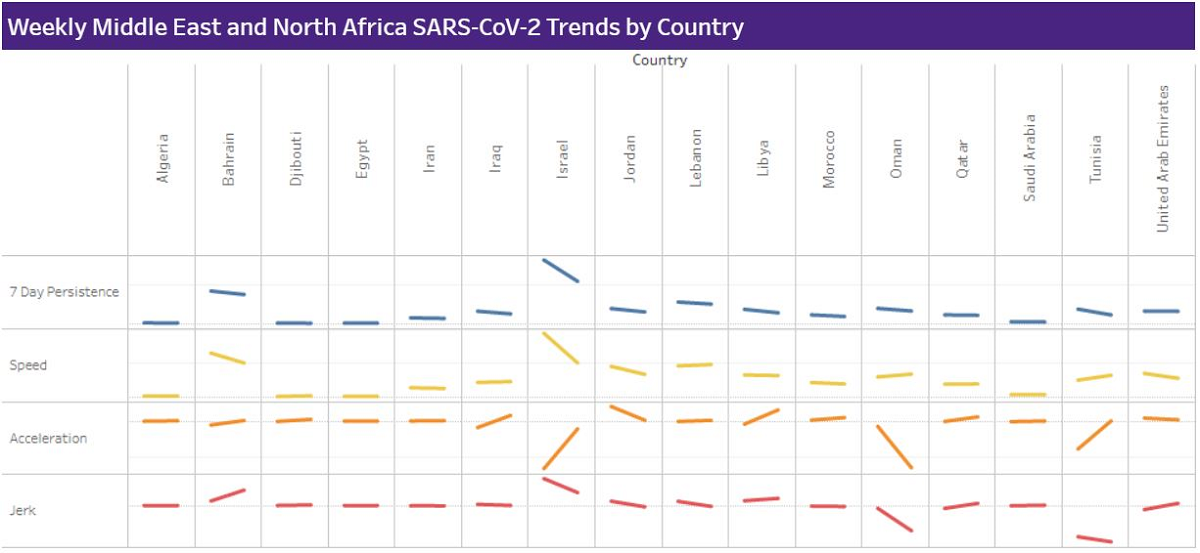

Supplement: Multimedia Appendix 1 [file jmir_v23i1e25830_app1.png]

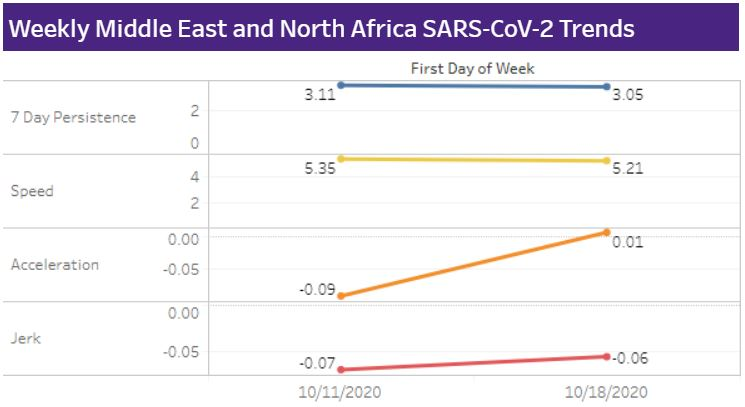

Supplement: Multimedia Appendix 2 [file jmir_v23i1e25830_app2.png]

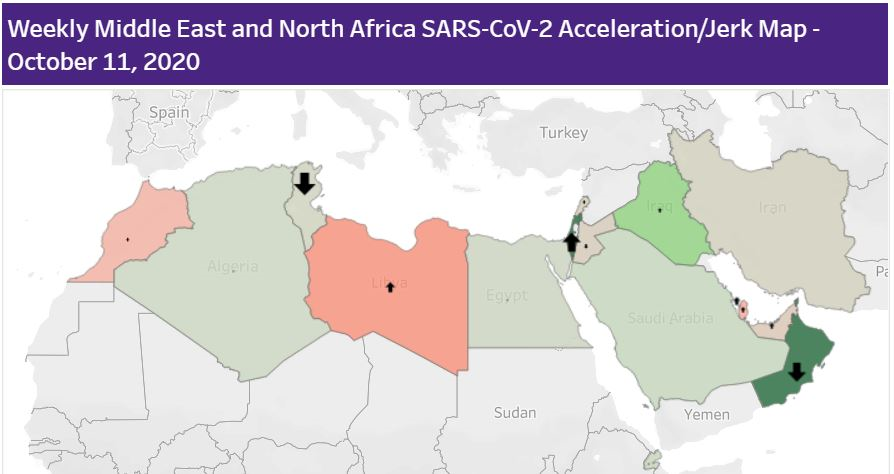

Supplement: Multimedia Appendix 3 [file jmir_v23i1e25830_app3.png]

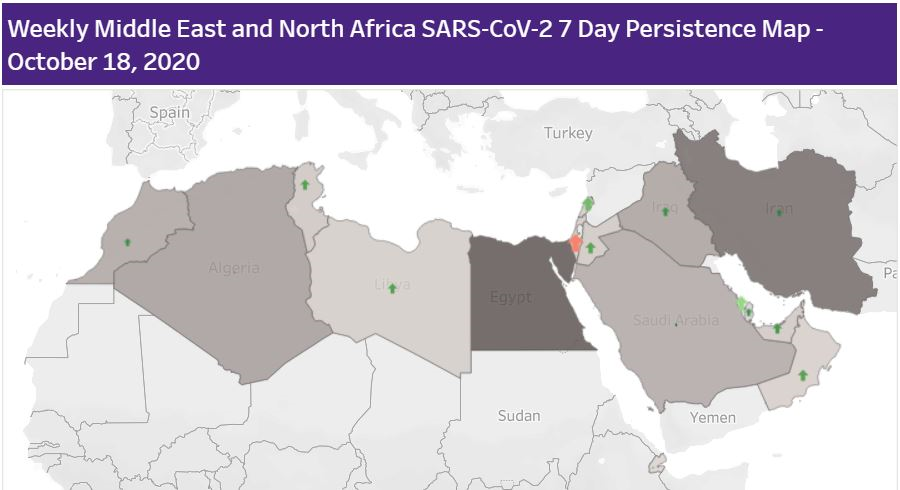

Supplement: Multimedia Appendix 4 [file jmir_v23i1e25830_app4.png]

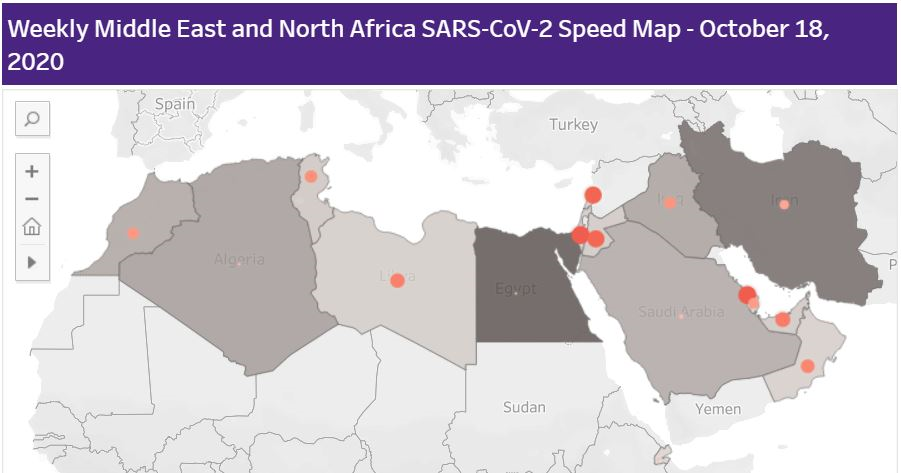

Supplement: Multimedia Appendix 5 [file jmir_v23i1e25830_app5.png]
